# “Humanizing intensive care: A scoping review (HumanIC)”

**DOI:** 10.1177/09697330211050998

**Published:** 2021-12-12

**Authors:** Monica Evelyn Kvande, Sanne Angel, Anne Højager Nielsen

**Affiliations:** 155319 Lovisenberg Diaconal University College, University Hospital of North Norway, Norway; 1006 Aarhus University, Denmark; 5562 Molde University College, Norway; 60169 Gødstrup Hospital, Aarhus University, Denmark

**Keywords:** Dehumanizing, humanizing, intensive care, intensive care unit, scoping review

## Abstract

Significant scientific and technological advances in intensive care have been made. However, patients in the intensive care unit may experience discomfort, loss of control, and surreal experiences. This has generated relevant debates about how to humanize the intensive care units and whether humanization is necessary at all. This paper aimed to explore how humanizing intensive care is described in the literature. A scoping review was performed. Studies published between 01.01.1999 and 02.03.2020 were identified in the CINAHL, Embase, PubMed, and Scopus databases. After removing 185 duplicates, 363 papers were screened by title and abstract. Full-text screening of 116 papers led to the inclusion of 68 papers in the review based on the inclusion criteria; these papers mentioned humanizing or dehumanizing intensive care in the title or abstract. Humanizing care was defined as holistic care, as a general attitude of professionals toward patients and relatives and an organizational ideal encompassing all subjects of the healthcare system. Technology was considered an integral component of intensive care that must be balanced with caring for the patient as a whole and autonomous person. This holistic view of patients and relatives could ameliorate the negative effects of technology. There were geographical differences and the large number of studies from Spain and Brazil reflect the growing interest in humanizing intensive care in these particular countries. In conclusion, a more holistic approach with a greater emphasis on the individual patient, relatives, and social context is the foundation for humanizing intensive care, as reflected in the attitudes of nurses and other healthcare professionals. Demands for mastering technology may dominate nurses’ attention toward patients and relatives; therefore, humanized intensive care requires a holistic attitude from health professionals and organizations toward patients and relatives. Healthcare organizations, society, and regulatory frameworks demanding humanized intensive care may enforce humanized intensive care.

## Introduction

Significant scientific and technological advances within intensive care units (ICUs) have been made, improving survival rates.^1,2^ Patients in the ICU have life-threatening illnesses and injuries that require monitoring and specialized treatment for survival.^3^ The clinical scenarios of ICU patients are complex and characterized by the potential for organ failure and death.^3,4^ A patient’s condition can shift rapidly from improvement to deterioration, and patients can descend into liminal states and face life-or-death situations,^5,6^ and several studies have shown that ICU patients may experience discomfort, panic, anxiety, unreal experiences, and dreams.^5–7^ In this situation, patients depend on nurses’ attention and ability to understand and respond to their emotions, symptoms, and needs.^8–10^

However, the occasional brutal realities of contemporary ICUs have generated relevant debates about how to humanize the ICU and whether humanization is necessary at all.^11,12^ Moreover, a wide variety of interventions have been suggested to humanize the ICU, ranging from improved visitation policies,^13–15^ practices to humanize the environment,^13,14,16–18^ improved communication,^16^ the implementation of diaries,^19^ and professional education and care models.^20^ Although much effort has been made to improve the perception of intensive care among patients, relatives, and healthcare professionals,^21^ humanizing intensive care remains veiled behind the multitude of interventions claiming to humanize the ICU. Consequently, a deeper understanding of what constitutes humanizing intensive care needs to be achieved.^22^ Therefore, this paper aims to explore how humanizing intensive care is described in the literature.

## Methods

The framework described by Arksey and O’Malley^23^ and further refined by others^24,25^ was applied in this scoping review and includes the following steps: identifying the research question; identifying the relevant studies; selecting studies; charting the data; collating, summarizing and reporting the results, and performing a consultation exercise.^23^ According to Arksey and O’Malley,^23^ the scoping review process is not linear but iterative, requiring researchers to engage with each stage of the research process in a reflexive way and, where necessary, repeat steps to ensure that the literature is covered in a comprehensive manner.

### Identification of relevant studies

In February 2020, a detailed search was performed in the CINAHL, Embase, PubMed, and Scopus databases for articles published between 01.01.1999 and 02.03.2020. We limited our scope to studies published since 1999, given that a paradigm shift in intensive care occurred with the use of light sedation, allowing patients to be more conscious and alert.^26,27^ In collaboration with a research librarian, we developed a search strategy consisting of both truncated keywords and medical subject headings. The main search terms used were intensive care unit, critically ill patients, critical care nursing, humanism, holistic nursing, and patient centered care. The search terms were combined with the Boolean operators “AND” and “OR” to both narrow and expand the search.

Any mentioning of *humanizing* or *dehumanizing intensive care* in the *title or abstract* was explored to find eligible literature for inclusion. This counted research article, conference abstract, editorial, or commentary. We chose to exclude literature focusing on end-of-life care, organ donation, pediatric or neonatal intensive care, laboratory studies, or settings outside intensive care were excluded to focus on ICU patients and their relatives simply being in the ICU receiving treatment and care. Literature not published in English, Norwegian, Danish, or Swedish were also excluded.

### Collating, summarizing, and reporting results

We uploaded our search results to Covidence, an internet-based application for handling, screening, and including papers in systematic reviews. After removing 185 duplicates, 363 papers were screened by title and abstract. Full-text screening of 116 papers led to the inclusion of 68 papers in the review. Screening was performed in Covidence, allowing two assessors to independently assess each paper for inclusion. In case of disagreement, a third assessor made the final decision. A priori, we decided to extract the following data from each included paper: authors, publication year, journal, title, country of origin, publication type, aim, methods, findings, or results. Data extraction and descriptions of humanized care from each paper were reviewed by a second assessor. The qualitative extraction of data was not supported by Covidence but was managed using an Excel spreadsheet.

We summarized the entire data set in terms of publication year, country of origin, and publication type. The description of humanized care was thoroughly reviewed and related to other studies to identify how humanized intensive care is described, definition of humanizing intensive care, words used to describe humanizing intensive care, and theoretical background for humanizing intensive care. During the analysis process, the authors continuously reflected on the findings and discussed their implications.

We consulted with two stakeholders within the ICU community to examine our approach to the review and they contributed additional valuable insights regarding our results. This process is in line with the recommendations of Arksey and O’Malley^23^ to enhance the meaning and applicability of the scoping review.

We used the Preferred Reporting Items for Systematic Reviews and Meta-analyses: The PRISMA Statement to construct the flow diagram.^28^

**Figure 1.**
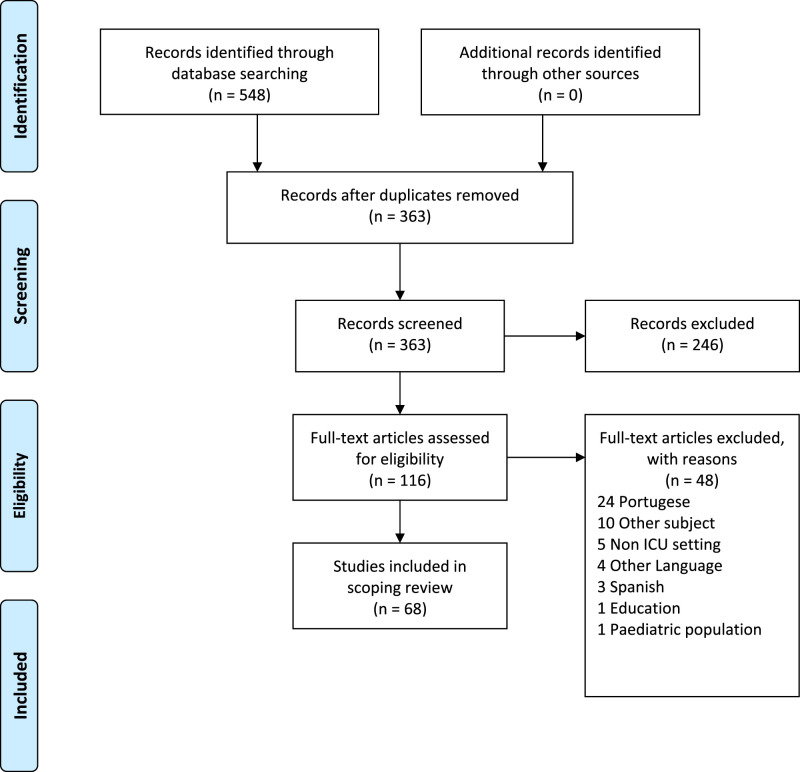
PRISMA flow diagram. From: Moher et al. (2009).

## Results

The included 68 unique articles included 32 empirical research papers, 10 abstracts from conferences, six editorials, six point-of-view papers, five systematic reviews, two essays, three posters, two commentaries, one theoretical paper, and 1 case study. Geographically, studies were from Brazil (15), the US (13), the UK (10), Spain (10), and Canada (7) with a few studies from Australia (4), Jordan (1), Iran (1), Denmark (1), France (1), Portugal (1), the Netherlands (1), Nepal (1), Ecuador (1), and New Zealand (1). The majority (42 of the 69) of the papers were published between 2015 and 2020 (see Tables 1 and 2).

**Table 1.** Type of paper by year published.

	1999	2000	2001	2002	2003	2004	2005	2006	2007	2008	2009	2010	2011	2012	2013	2014	2015	2016	2017	2018	2019	Total
Empirical research	1			1		2			1	2	2				3	2	2	5	1	3	7	32
Abstracts from conferences											1	2				1	1		2		3	10
Editorials				1			1												2	1	1	6
Point-of-view papers																1	1	2		1	1	6
Systematic reviews/reviews			1															1		3		5
Essays				1				1														2
Posters	1																1				1	3
Commentaries																			1	1		2
Theoretical papers								1														1
Case study														1								1
Total	2	0	1	3	0	2	1	2	1	2	3	2	0	1	3	4	5	8	6	9	13	68

**Table 2.** Type of paper by country of origin.

	Australia	Brazil	Canada	Denmark	Ecuador	France	Iran	Jordan	Nepal	New Zealand	Netherlands	Portugal	Spain	UK	US	Total
Empirical research	1	12	3	1	1	1	1	1	1				1	7	2	32
Abstracts from conferences		1	1									1	1	2	4	10
Editorials	1												2		3	6
Point-of-view papers	1										1		4			6
Systematic reviews/reviews		1	1												3	5
Essays	1									1						2
Posters			2												1	3
Commentaries													2			2
Theoretical papers														1		1
Case study		1														1
Total	4	15	7	1	1	1	1	1	1	1	1	1	10	10	13	68

Humanizing intensive care was defined as holistic care of the patient, a general attitude of professionals toward the patients and relatives, or an organizational trait toward all subjects of the healthcare system. Technology was seen as an integral part of intensive care that needed counterbalancing by humanistic care. The large number of studies from Spain and Brazil reflected the strong and growing interest in humanizing intensive care in these particular countries.

### Defining humanizing care

This scoping review revealed that a consensus on what humanized care is and how it is described is lacking. All included papers were reviewed for explicit descriptions of humanizing care. Twelve papers took steps to define humanization in intensive care.^11,13,14,17,29–36^ Two of the 12 papers drew on the Royal Spanish Academy’s definition of humanization as an effort “to make someone human, familiar and affable,”^11,17^ whereas the remaining 10 papers revolved around three main themes: “humanizing care as holistic care of the patient”; “humanizing care as a general attitude of professionals toward the patients”; and “humanizing care as an organizational trait toward all subjects of the healthcare system.”

### Humanizing care as holistic care of the patient

Some papers described humanizing care as being holistic,^14,32,35^ which implied seeing the patient as a whole^35^ and taking into account both the family and social context.^32^ Although not using the word holism in relation to humanized care, other papers point in the same direction by stressing that the patient is not only a biological entity but also an autonomous person^32^ with psychosocial and spiritual needs^30,34^ and inherent ethical dignity.^34^ Humanized care encompasses that relatives are met as whole, autonomous persons as well.^31^ Moreover, families should be encouraged to play an active part in the team around the patient,^14,18^ and the wellbeing and autonomy of families should be a healthcare goal.^14,35,36^

### Humanizing care as a general attitude at an interpersonal level

Humanizing care respects and preserves the patient’s human dignity.^31,33,34^ The act of humanizing care must come from health professionals, as suggested by Heras La Calle et al.,^29^ Heras La Calle et al.,^30^ and Mondadori et al.^33^ It must be a personal and collective vow to humanize the healthcare system by promoting health in all aspects of clinical practice,^29,30,33^ including prevention, care, rehabilitation and end-of-life care,^33^ as well as relationships, behaviors, and the environment.^29^ This implies understanding oneself as part of an intricate and complex system spanning policies, management, and culture, all of which influence the ability to place the patient at the center of health care.^30^ Good communication among management, healthcare professionals, patients, and relatives is viewed as essential to achieve a humanized attitude toward patients.^33,36^

### Humanizing care: An organizational trait favoring all its subjects

On an organizational level, healthcare systems are described by Heras la Calle et al., as humanized when they place themselves at the service of patients, relatives, and healthcare professionals.^30^ Serving the individual implies that organizations are aware of subjective vulnerabilities^29,36^ and actively try to protect vulnerable patients, relatives, and healthcare professionals by coping with power asymmetries and dehumanizing attitudes.^36^ To minimize dehumanization, healthcare systems should strive to create environments and working conditions^30,32,37^ that enable healthcare professionals to engage with patients in high-quality^30,34^ multiprofessional health care.^14,35^

### Emotional distancing by dehumanizing patients may protect professionals

Dos Santos et al.,^38^ García Martínez et al.,^39^ and Lopes and Brito^31^ underlined healthcare professionals’ attitudes as key to humanizing intensive care for patients. However, patients continue to be referred to in terms of diagnoses, tasks, or organ failures.^12,40^ In a 2015 editorial in *Intensive Care Medicine*, Kompanje et al.^12^ argue that dehumanizing patients by referring to them as things serves a distinct purpose: it protects physicians from feeling the pain of patients and relatives and allows them to go on working in the intensive care unit over time.^12^ This is supported by McLean et al.,^40^ who argue that different ways of talking about patients serve different purposes. Specifically, talking about the patient in terms of organ failure may help the nurse analyze the situation, whereas talking about the patient in terms of tasks may help the nurse prioritize their time. In addition, Pereira et al.^34^ suggest that the conflict between reality and ideality may cause nurses to experience emotional distress and burnout. This finding is similar to the findings of McLean et al.,^40^ who demonstrated that nurses reported frustration and moral distress when they failed to care for the “whole person.” Additionally, moral conflicts as a source of burnout in healthcare professionals corresponds well with the views of Kompanje et al.,^12^ who also note the difficulties associated with the emotional involvement and suffering of patients and relatives. However, Kompanje et al.^12^ stress that it is sufficient for healthcare professionals to know that their patients and relatives suffer and act to relieve this suffering.^12^ In this context, the actual actions of healthcare professionals are key to humanizing the care of patients and relatives in the ICU.

### Mastering and transcending all-present technology to provide humanized care

Technological developments in health care have contributed to the reduction of morbidity and mortality, increased life expectancy, and optimized technical activities in the ICU. However, this development has not secured care focused on human values.^34^ Rather, de la Fuente-Martos et al.^17^ stated that one main cause of dehumanization is the intensive use of technology and the “dictatorship of technology,” which leads to patients being seen as “things.” Nevertheless, Wilkin and Slevin^41^ found that technological competence was an essential part of ICU nursing despite the notion that nurses cite that technology to conflicts with humanistic care. This is supported by the findings of Alasad,^42^ namely, that the use of technology and machinery in routine intensive care is considered demanding and time-consuming despite making nurses feel safe and in control. However, Alasad^42^ also found that when nurses’ technological competencies increased, their attention shifted toward the patient and family while the equipment faded into the background. Moreover, nurses were valued for supporting the patient. Therefore, nurses need to balance humanistic caring and technology.^41,43^ This balancing requires nurses to be experienced and therefore able to prioritize care for the individual patient.^41^ Alliex and Irurita^44^ found that experienced nurses manipulated technology and the environment when they thought it was for the benefit of the patient, for example, decreasing alarms or protecting the patient from lights and sounds to keep the patient relaxed.

Additionally, patients had a nuanced perspective on technology as both alienating and reassuring, uncomfortable but comforting, and impersonal yet personal.^45^ The patients found that nurses could minimize the alienating effect of technology by maintaining a close and supportive presence and providing comfort and care.^45^ The call for presence and personal care is supported by McCallum,^43^ who suggests human touch, being present and using patient names as key features of humanistic caring used to ameliorate the negative consequences of technology.^43^ Consequently, nurses need to master technology to transcend it and thus be able to focus on the patient as a whole, not just as an extension of the technological equipment.^46^ To facilitate this transcendence, da Silva and Ferreira^47^ claim that it is essential that the use of technology in intensive care should be guided by humanistic principles such as respect for the uniqueness of the individual and human dignity. As a result, intensive care nursing performed in the ICU environment is expected to combine technique, technology, and humanizing care.

### The Brazilian and Spanish movements toward humanizing intensive care

Our literature search excluded 39 Brazilian papers and 12 Spanish papers not published in English and, therefore, inaccessible to us. Nonetheless, this large number of studies adds to our finding of a strong and growing interest in humanizing intensive care in Spain and Brazil. We included 15 papers from Brazil.^20,31–34,36,38,47–54^ Most of the Brazilian papers included in this review referred to either the National Humanitarian Assistance Program (PNHAH) launched by the Brazilian Ministry of Health in 2001^20,32–34,36,48–51^ or one of the several editions of the Brazilian National Humanization policy launched as early as 2004.^36,48^ The recurring references to national policies and programs indicate that a national political interest in promoting humanism in health care may have a tremendous effect on health care and function as a framework for healthcare research. However, Lugarinho et al.^54^ describe a program of humanizing intensive care as early as 1996, which is 5 years prior to the founding of the Brazilian National Humanitarian Assistance Program.^36^ Thus, a nascent trend toward humanized health care within intensive care preceded the national initiatives launched by the Brazilian Ministry of Health.

We included 10 papers from Spain.^11,14,17,29,30,35,55–58^ These 10 Spanish papers were initiated by an editorial by Kompanje et al.^12^ in *Intensive Care Medicine* in 2015. Quite boldly, the authors ask whether the ICU is a branch of Hell as claimed by a former patient on national TV. In 2014, the influential Spanish research project aiming to humanize intensive care (Proyecto HU-CI) was launched.^30^ This research initiative highly impacted the literature included in this review. Of the 10 included papers originating from Spain, 4 papers stemmed directly from the HU-CI project^14,29,30,55,^ and three others refer to the HU-CI project.^17,35,58^ One paper raises some form of critique of the humanizing movement and the proposed need to change healthcare professionals but acknowledges that humanized intensive care benefits patients.^11^

## Discussion

In reviewed papers, we found that humanized care was defined in terms of holistic care of patients and relatives, as a professional attitude toward patients and as a capacity of the healthcare organization. Technology has risk to dehumanize patients; therefore, it is essential that healthcare professionals proficiently master technology, allowing them to see beyond technology to the patient as a human being. To prevent emotional burnout, healthcare professionals may distance themselves from the patient; however, if patient suffering is acknowledged and addressed, humanized care may be provided. Geographical differences were noted. The humanizing care movement seemed particularly strong in Spain and Brazil sparked by research and political initiatives, whereas papers describing humanizing care in other parts of the world were lacking. However, this lack of publications does not mean that a similar discussion is not warranted or occurring; rather, it may indicate that other concepts, such as patient-centered care, patient involvement, or patient wellbeing, have been in focus.

Patient-centered care has been defined as the provision of care consistent with the patient’s values, needs, and desires. To achieve patient-centered care, health professionals must involve patients in decisions on health care.^59^ While patient involvement is necessary for the provision of patient-centered care, patient involvement may reflect a wish to encourage consumerist thinking among those who receive care and a wish for patients to take a more active part in their own care.^60^ A taxonomy on patient involvement developed by Thompson^60^ linked the level of patient involvement to the level of patient power suggesting that some patients may not have the energy to be involved in healthcare decisions. A scoping review by Olding et al.^61^ supports this and shows that more research is needed to understand how patient involvement in the ICU can be practiced given the limitations ICU patients may experience. Thompson^60^ identified patient involvement to be codetermined by healthcare professionals and patients. This concept corresponds with the findings of this review, which has identified the attitudes of professionals as pivotal to achieving humanized care.

This review showed that humanized care was often defined as holistic care, which was described in terms of seeing the patient as a whole and taking into account psychosocial and spiritual needs, but no consistent definition of holism was adhered to. Supporting this notion is an editorial by Bullington and Fagerberg^62^ that refers to holistic care “a fuzzy concept” and argues that more research is needed. A recent study by Jasemi et al.^63^ identified holistic care and patient-centered care as two concepts used interchangeably. However, Jasemi et al.^63^ proposed that holistic care should be defined as care for all of the patients’ needs, considering the patient as unique being with the primary objective to provide comfort. Moreover, holistic care should not be limited to the patients’ needs. Importantly, their culture and spiritual needs should be taken into account and holistic care should improve conditions for both patients and healthcare personnel.^63^ Consequently, the definition proposed by Jasemi et al.^63^ goes well beyond patient-centered care and may be compared to defining humanizing care as an organizational trait toward patients, relatives, and healthcare professionals. Moreover, it shows that the provision of holistic care is closely linked to proficiency and personal growth in healthcare professionals. A similar concept is found in this review, where the importance of overcoming the dehumanizing effects of technology is closely related to nurses’ competencies that allow them to manage and look beyond technology to see patients as fellow human beings.

Todres et al. propose lifeworld perspectives as a “humanizing force” to moderate the grip technological progress holds to processes, systems, and institutions of health care.^64^ Lifeworld-led health care seeks to understand what meaning the situation holds to the unique person, provide language to this understanding, and use it to underpin health care for that person.^64^ Relating this to the findings of this review, humanized care involves seeing the patient and relative as fellow human beings, providing attention to their needs and their situation and expressing this attention in action and interaction between patients, relatives, healthcare professionals, and healthcare organizations.

### Strengths and limitations

Stakeholder consultations validated and nuanced our findings reminding us to broaden our findings to all patients in the ICU regardless of their level of consciousness and ability to communicate with healthcare professionals. Furthermore, stakeholders agreed that acknowledging patients over technology was important to humanize care in the ICU, but that the healthcare organizations including working conditions should be viewed as an equal contributor to humanized care in the ICU.

In this scoping review, a major limitation may be the language inclusion criteria (English and Scandinavian) as our initial search showed a large body of literature in Portuguese and Spanish that was excluded. Nevertheless, we believe that the included papers mirror the general trend in the literature on humanizing intensive care in Spain and Brazil. We did not appraise the quality of evidence of the included studies but rather presented how humanizing intensive care is described in the variety of studies.

### Implications and recommendations for practice

We recommend that nurses and other healthcare professionals, organizations, and societies work for holistic and humanized intensive care that involves seeing the patient and relative as fellow human beings, providing attention to their needs and their situation, and expressing this attention in action and interaction between patients, relatives, healthcare professionals, and healthcare organizations. To achieve this, healthcare professionals must receive appropriate training on technology equipment to balance the lifesaving effects of technology with respect for human dignity.

## Conclusion

A multitude of perspectives on humanizing intensive care, especially from the Spain and Brazil, reflects a growing interest in humanizing intensive care. To achieve humanized intensive care from the patients’ and relatives’ perspectives, a holistic view of the patient must be reflected in the attitudes of nurses and other healthcare professionals. This implies balancing the lifesaving effects of technology with a greater emphasis on the individual patient and respect for human dignity. Furthermore, humanized intensive care may be enforced by healthcare organizations by establishing humanizing conditions for patients, relatives, and healthcare professionals and by society by setting up a regulatory framework demanding humanized care for patients in the ICU.
